# Risk Factors and Prevention of Cancer and CVDs: A Chicken and Egg Situation

**DOI:** 10.3390/jcm14093083

**Published:** 2025-04-29

**Authors:** Maurizio Giuseppe Abrignani, Fabiana Lucà, Vincenzo Abrignani, Mariacarmela Nucara, Daniele Grosseto, Chiara Lestuzzi, Marinella Mistrangelo, Bruno Passaretti, Carmelo Massimiliano Rao, Iris Parrini

**Affiliations:** 1 O.U. of Cardiology, P. Borsellino Hospital Marsala, ASP Trapani, 91025 Marsala, Italy; 2 O.U. Interventional Cardiology-ICCU, A.O. Bianchi Melacrino Morelli, 89128 Reggio Calabria, Italy; maricanucara@gmail.com; 3 Internal Medicine and Stroke Care Ward, Department of Health Promotion, Mother and Child Care, Internal Medicine and Medical Specialties, University of Palermo, 90133 Palermo, Italy; maur.abri@alice.it; 4 Cardiology Unit, Hospital Ceccarini, 47838 Riccione, Italy; daniele.grosseto@auslromagna.it; 5 Esperia Medical Center, 33080 Porcia, Italy; chiara.lestuzzi@gmail.com; 6 Department Rete Oncologica Piemonte e Valle d’Aosta, Città della Salute e della Scienza, 10126 Turin, Italy; mmistrangelo@reteoncologica.it; 7 Cardiology Unit, Homanitas, Gavazzeni-Castelli, 24125 Bergamo, Italy; bruno@passaretti.org; 8 Cardiology Unit, Hospital Santa Maria Degli Ungheresi, 89024 Polistena, Italy; 9 Cardiology Department, Ospedale Mauriziano Umberto I, 10128 Turin, Italy; irisparrini@libero.it

**Keywords:** cancer, cardiovascular disease (CVD), risk factors, prevention

## Abstract

Cardiovascular diseases and cancer are the two primary causes of mortality worldwide. Although traditionally regarded as distinct pathologies, they share numerous pathophysiological mechanisms and risk factors, including chronic inflammation, insulin resistance, obesity, and metabolic dysregulation. Notably, several cancers have been identified as closely linked to cardiovascular diseases, including lung, breast, prostate, and colorectal cancers, as well as hematological malignancies, such as leukemia and lymphoma. Additionally, renal and pancreatic cancers exhibit a significant association with cardiovascular complications, partly due to shared risk factors and the cardiotoxic effects of cancer therapies. Addressing the overlapping risk factors through lifestyle modifications—such as regular physical activity, a balanced diet, and cessation of smoking and alcohol—has proven effective in reducing both CV and oncological morbidity and mortality. Furthermore, even in patients with established cancer, structured interventions targeting physical activity, nutritional optimization, and smoking cessation have been associated with improved outcomes. Beyond lifestyle modifications, pharmacological strategies play a crucial role in the prevention of both diseases. Several cardiovascular medications, including statins, aspirin, beta-blockers, and metformin, exhibit pleiotropic effects that extend beyond their primary indications, demonstrating potential anti-neoplastic properties in preclinical and observational studies. Recently, novel therapeutic agents have garnered attention for their possible cardioprotective and metabolic benefits. Glucagon-like peptide-1 receptor agonists (GLP-1 RAs) and sodium-glucose cotransporter-2 inhibitors (SGLT2is), initially developed for managing type 2 diabetes, have shown CV and renal protective effects, alongside emerging evidence of their role in modulating cancer-related metabolic pathways. Inclisiran, a small interfering RNA targeting PCSK9, effectively lowers LDL cholesterol and may contribute to reducing CV risk, with potential implications for tumor biology. Additionally, sacubitril/valsartan, an angiotensin receptor–neprilysin inhibitor, has revolutionized heart failure management by improving hemodynamic parameters and exerting anti-inflammatory effects that may have broader implications for chronic disease prevention. Given the intricate interplay between CVD and cancer, further research is essential to clarify the exact mechanisms linking these conditions and assessing the potential of CV therapies in cancer prevention. This review aims to examine shared risk factors, consider the role of pharmacological and lifestyle interventions, and emphasize crucial epidemiological and mechanistic insights into the intersection of CV and oncological health.

## 1. Introduction

Cardiovascular disease (CVD) and neoplasms persist as the two predominant causes of mortality globally, notwithstanding notable advancements in preventive strategies, early detection, and innovative therapies, improving the prognosis [1,2,3]. In addition, there is a bidirectional link between these two diseases [4,5].

Notably, epidemiological studies indicate a heightened prevalence of cancer among individuals with cardiovascular disease [6,7]. Patients with CVD exhibit an increased risk of developing malignancies, which may be attributed to shared risk factors, chronic inflammation, and the long-term effects of cardiotoxic treatments [6,8]. Recent findings suggest that cancer incidence is significantly elevated in individuals with a history of acute myocardial infarction (AMI), heart failure (HF), or other cardiovascular (CV) conditions, underscoring the necessity of interdisciplinary management approaches to optimize patient outcomes [4,6].

In regions where healthier lifestyles prevail, such as developed nations, there is a reduced incidence of both CVD and cancer, resulting in a noteworthy 30% decline in mortality attributed to specific cancer types. Further prevention strategies could be attained by addressing modifiable risk factors, including smoking, obesity, alcohol consumption, inadequate dietary patterns, sedentary behaviors, and pollution, which, as per recent findings, contribute to the onset of both maladies.

Significantly, inflammation acts as a vital link among these risk factors, CVD, and neoplasms.

Inflammation plays a crucial role in the development of atherosclerosis, functioning throughout all its phases, from leukocyte adhesion to the vessel wall to cytokine production by macrophages and monocytes, ultimately leading to thrombotic events. Furthermore, chronic inflammatory states underpin approximately 10–20% of tumor occurrences, potentially acting as both a causative agent and a consequence, thereby promoting disease progression and immune suppression. Persistent inflammatory conditions arise from various factors, resulting in chronic immune activation, excessive secretion of pro-inflammatory cytokines, and sustained oxidative stress. This prolonged interplay of cellular injury creates a microenvironment that favors cancer development. Infections also contribute, accounting for 8% of tumors, with documented associations between Papillomavirus and cervical neoplasms, Epstein–Barr virus and lymphoproliferative lesions in cerebral and oral tissues, Helicobacter pylori and gastric neoplasms and lymphomas, as well as hepatitis B and C viruses and hepatocellular carcinoma.

## 2. CV Risk Factors and Cancer

Cardiovascular (CV) risk factors, such as tobacco consumption, obesity, physical inactivity, and inappropriate diet are, at the same time, risk factors for tumor incidence [9]. It has been shown that cancer disability-adjusted life years (DALYs) might be significantly reduced by modifying risk factors, including smoking, obesity, alcohol use, and pollution [10,11,12]. Obesity is the most common risk factor (67%), followed by a lack of physical activity (24%) and alcohol consumption (16%) [13,14]. In a two-sample Mendelian randomization analysis, lung cancer has been associated with poor physical activity and smoking [15,16,17,18,19].

Cigarette smoking is the most significant cause of preventable death, responsible for approximately one-third of cancer-related deaths. It is well established that the carcinogenic substances in cigarette smoke promote the development of lung tumors (smoking is responsible for 90% of cases of this neoplasm) [20]. However, cigarette smoking is also associated with oral and throat cancer, pancreatic cancer, colon cancer, bladder cancer, kidney cancer, esophageal cancer, breast cancer (especially among younger women), and certain leukemias [21].

Although the causal relationship between cigarette smoking and oncogenesis is well established, the underlying pathogenetic mechanisms of this correlation have only recently been described. Cigarette smoke introduces more than 7000 chemical agents into the body, at least 70 of which have been identified as carcinogens. Several studies published in recent years have demonstrated that smoking approximately twenty cigarettes per day for a year causes around 150 genetic mutations in each lung cell. There are at least five mechanisms through which smoking damages DNA, leading to mutations that intrinsic repair mechanisms cannot repair.

Diet has been recognized as an essential risk factor for many types of cancer, particularly for those of the digestive sites [22]. Glycemic values have been significantly associated with kidney [17], stomach, colorectal cancer [18,19,23], and pancreatic cancer [24].

**Figure 1:**
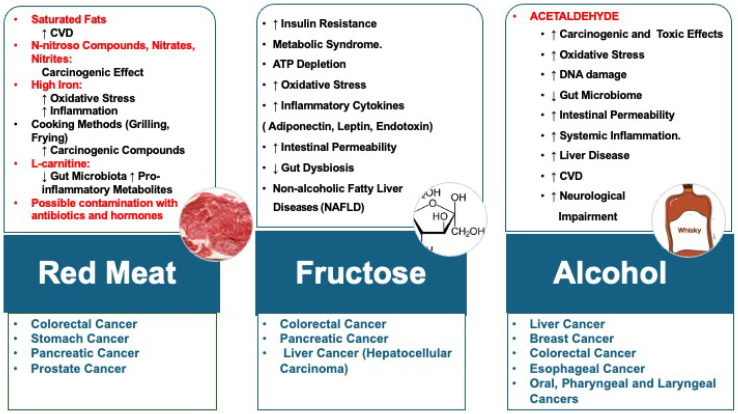
Adverse health impacts of certain foods. Legend: CVD: cardiovascular diseases. ↓: REDUCTION; ↑: INCREASE.

The recognition of nutrition’s pivotal role in cancer incidence and progression has been longstanding [25,26] (Figure 1). Notably, diets rich in animal proteins have been associated with elevated mortality rates [27,28,29]. However, it is essential to underscore that processed red meat, in particular, is associated with a markedly adverse impact on cancer mortality. The factors contributing to the deleterious effects of red meat, especially when processed, are manifold [30,31]. Oxysterols, oxygenated derivatives of cholesterol prevalent in cholesterol-rich foods, like raw meat and pork loin, have been implicated in modulating numerous physiological processes and cellular functions, thereby playing a significant role in conditions such as atherosclerosis, diabetes mellitus, and prostate, breast, colon, and bile duct tumors [32].

Notably, the composition of the Western diet, which is rich in animal proteins and saturated fatty acids while low in fiber, has been shown to alter the gut bacterial ecosystem unfavorably [33,34].

Furthermore, the adverse impact of animal-based diets on tumor development is compounded by environmental pollution. Livestock farming accounts for significant methane (37%), nitrous oxide (65%), and ammonia (64%) emissions, thereby exacerbating the greenhouse effect and contributing to acid rain. Alcohol use is significantly related to cancer [35,36]. When combined with smoking, the risk of alcohol increases further; this synergy effect was evident, especially for head and neck cancer [37].

Physical inactivity has been considered a cancer risk factor [38,39].

According to International Agency for Research on Cancer (IARC) data, a high BMI index may be the cause of up to half a million cancer cases per year. It has been established that there are significant associations between obesity and breast malignancy (postmenopausal), urologic cancers, and pancreatic ductal adenocarcinoma. Obesity is also linked to an increased risk of for esophagus/stomach, prostate, liver, colon–rectum, endometrium, and kidney cancers, as well as leukemia [40,41,42,43,44,45,46,47,48,49], including childhood leukemia [50,51,52,53,54]. Obese individuals commonly exhibit heightened levels of estrogen, particularly noteworthy in postmenopausal women, correlating with the occurrence of breast and endometrial tumors [55].

Similarly, obesity exhibits a substantial correlation with CV risk [56,57].

Visceral adipose tissue, endowed with metabolic, endocrine, and immunological functions, is pivotal in metabolic syndrome and cancer progression genesis [58].

Diabetes contributes to macro- and microvascular injury, fostering the onset of CVDs. A comprehensive meta-analysis delineated pronounced correlations between diabetes and specific cancer types, including breast, liver, pancreas, colon/rectum, endometrium, and bladder malignancies [59,60].

From a mechanistic standpoint, diabetes and CVD share intertwined pathophysiological mechanisms. Insulin resistance, which underpins inflammation, precipitates dyslipidemia, characterized by elevated levels of atherogenic, very low-density lipoproteins and triglycerides [61]. Hyperglycemia, hyperinsulinemia, and heightened IGF-I levels stimulate smooth muscle cell migration and proliferation, which underpin atherosclerosis. They concurrently activate insulin receptors on tumor cells, thereby fostering their proliferation and dissemination [58]. Hyperglycemia further compromises nitric oxide synthesis, thereby fostering endothelial dysfunction mediated by free radical release [62].

**Figure 2:**
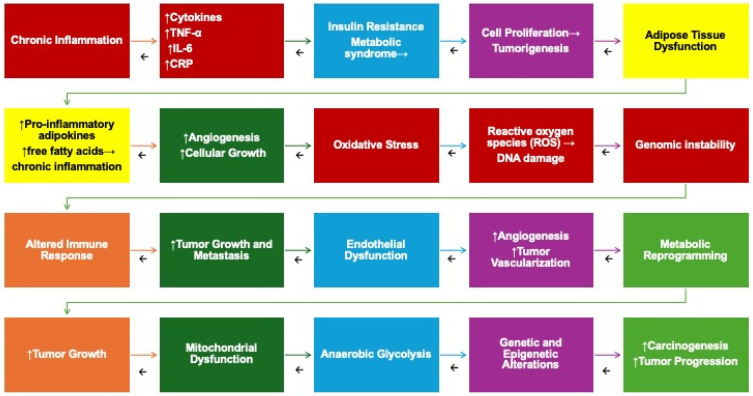
Pathophysiological mechanisms correlating inflammation, metabolic syndrome, and neoplasms. Legend: IGF1: Insulin-like growth factor, IL6: interleukin-6, SHBG: sex hormone-binding globulin, TNFα: tumor necrosis factor-α. The enlargement of adipose tissue triggers the infiltration of macrophages and T cells into adipocytes, releasing proinflammatory cytokines and alterations in circulating adipokine levels. This process fosters insulin resistance, primarily affecting metabolic tissues, resulting in hyperinsulinemia and increased insulin-like growth factor 1 (IGF-1) synthesis. These metabolic disruptions not only underlie the development of metabolic syndrome but also play a significant role in promoting tumor initiation and progression. Additionally, bioactive molecules secreted by macrophages and adipocytes exert autocrine and paracrine effects, further exacerbating the inflammatory response.

Intricate interplay among hyperinsulinemia, inflammation, and neoplasms has been reported [63,64,65] (Figure 2).

**Figure 3:**
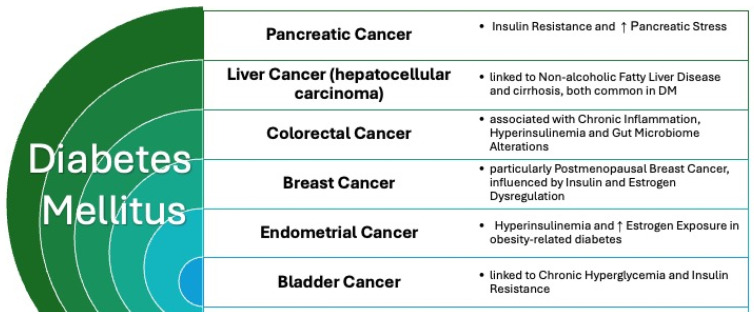
Cancers most commonly associated with diabetes.

The mechanisms connecting diabetes and cancer include increased insulin and IGF-1 signaling, which promotes cell proliferation, reduced apoptosis, and enhanced tumor growth. Additionally, chronic inflammation and oxidative stress in DM contribute to DNA damage and carcinogenesis. The cancers most commonly associated with DM are shown in Figure 3.

Although arterial hypertension represents a well-recognized CV risk factor, its pathophysiological linkage to cancer remains less elucidated. Hypertension exhibits a robust association with renal tumors and elevates the risk of colon–rectum, bladder, pharynx, melanoma, pancreas, and cervical neoplasms [66]. Putative mechanisms encompass vascular oxidative stress and a pro-inflammatory milieu. Elevated angiotensin-II levels elicit vasoconstriction via endothelial growth factor stimulation, promoting neoangiogenesis and tumor development [67,68].

Dyslipidemia is a significant risk factor for carcinogenesis, invasion, and metastasis. Atherosclerosis commences with excess lipoproteins, notably atherogenic low-density lipoproteins, infiltrating the vessel wall. Intracellular cholesterol assumes a pro-angiogenic role and exerts critical regulatory functions in carcinogenesis and metastasis. A cholesterol metabolite, 25-hydroxycholesterol, structurally akin to estradiol, appears implicated in breast cancer pathogenesis [69]. Elevated levels in tumor cell macrophages likely contribute to the underlying inflammatory milieu [70].

Psychological stress may exert an etiological influence on selected neoplasms, particularly reproductive tumors, colorectal cancer, melanomas, angiosarcomas, and multiple myelomas. This effect may be mediated by oxidative stress or sympathetic nervous system hyperactivity [71,72,73].

## 3. Tumors in Patients with CVD

In recent years, there has been discernible evidence indicating a heightened prevalence of neoplasms among individuals afflicted with cardiac conditions compared to the general population [74]. A Danish registry study disclosed that within instances of newly diagnosed heart failure (HF), the incidence of neoplasms escalates by 70% for two years [75]. Additionally, Banke’s research corroborates an augmented susceptibility to various cancer types, except prostate cancer, among HF patients [76].

Despite the elusive mechanistic underpinnings, it is conceivable to hypothesize that inflammation may influence both pathologies’ development, given that chronic inflammation and neurohormonal activation characterize HF [77].

## 4. Primary Prevention of Malignancies

A large body of evidence suggests that interventions aiming to reduce CV risk may contextually also reduce oncologic risk.

A diet abundant in plant proteins, vegetables, fruits, fish, and whole grain products exhibits a protective effect [78,79]. Polyunsaturated fatty acids (PUFAs) have also been reported to have a protective effect [80,81]. The role of fructose as an alternative sweetener remains contentious. Low doses are associated with glycemic control benefits, while higher consumption levels may lead to adverse metabolic outcomes, such as overweight, dyslipidemia, hyperuricemia, and insulin resistance [82].

Moreover, a comprehensive examination of the role of diet in preventing CVDs and neoplasms necessitates an analysis beyond individual food items. Instead, it must encompass an evaluation of various dietary patterns, defined as the combination of regularly consumed foods that synergistically influence diverse cardiometabolic risk factors [83]. Dietary quality scores have thus been devised to gauge diet quality, with improved scores associated with reduced all-cause mortality, notably a diminished risk of colorectal cancer mortality. The beneficial effects of vegetarian and Mediterranean diets on various cancer types are well documented, albeit influenced by factors such as smoking, age, menopause, gender, and body mass index [84,85,86]. However, it is essential to clarify the concept of the Mediterranean diet, as recent reports have highlighted a concerning rise in diabetes and obesity rates even in regions traditionally associated with the Mediterranean diet. Thus, adherence to the Mediterranean diet does not inherently equate to healthy eating. Instead, specific dietary guidelines must include whole grains, moderate wine consumption, avoidance of excessive alcohol, limited salt and sweets intake, and adherence to appropriate caloric intake to prevent overweight and obesity [87].

The evidence supporting the association between low-glycemic-index and low-glycemic-load diets and a reduced risk of developing various chronic diseases, including specific cancer types (notably colorectal and endometrial cancers), lends credence to the hypothesis that high postprandial glycemia may represent a global mechanism driving disease progression [88].

An area of particular interest in evaluating different dietary patterns pertains to the interplay among diet, immunity, and inflammation, especially considering certain foods’ ability to influence gut microbiota composition. Given the gut microbiota’s role as an endocrine organ, generating bioactive metabolites capable of affecting the host's intestine physiology, dietary choices can profoundly impact conditions such as HF, atherosclerosis, and cancer [89]. Recent observations suggest that gut microbiota composition may influence the immune system, with significant implications for tumor immune response. Diets rich in whole grains have been found to reduce systemic inflammatory status without disrupting gut microbiota composition [90].

In summary, diet’s impact on cancer prevention and progression is multifaceted, with various dietary patterns and food choices exerting differential effects on cancer risk. Adopting dietary practices that are conducive to overall health and well-being is imperative, informed by robust scientific evidence and tailored to individual needs and risk factors [91].

During different stages of life, physical activity is essential for properly developing and maintaining the musculoskeletal system and is beneficial for reducing stress, anxiety, and depression. Additionally, it plays a role in lowering the risk of developing chronic degenerative conditions, such as hypertension, diabetes, CVDs, and certain cancers [92,93].

A recent study found that moderate to intense physical activity levels were associated with a reduced incidence of 13 out of 26 types of tumors (ranging from 10% to 42%) [94]. Overall, the risk reduction for all considered tumors is approximately 7% [95].

Regular aerobic physical activity is believed to reduce the risk of tumor development by improving tissue oxygenation, thereby decreasing inflammatory status and eliminating accumulated toxic substances. Specifically, certain molecules called “myokines” (such as calprotectin) seem capable of enhancing apoptosis and reducing the activity of metalloproteinases involved in metastatic processes [96]. Movement also accelerates intestinal transit, limiting the contact time between potentially toxic waste substances from food and the gastrointestinal mucosa. Physical activity ensures better modulation of insulin plasma levels, improves tissue sensitivity to insulin, reduces levels of sex hormones involved in cell growth, optimizes immune system activity, and acts on energy metabolism, reducing the risk of overweight and obesity.

The World Cancer Research Fund (WCRF) has continuously updated specific recommendations on adequate physical activity [97]. At the same time, the European Code Against Cancer has further emphasized the importance of physical activity as a protective factor [98]. The World Health Organization (WHO) recommends that adults engage in daily physical activity, starting with a minimum of 30 min of moderate exercises (such as walking, slow swimming, or cycling) and gradually increasing the duration to achieve at least 60 min of moderate activity or at least 30 min of vigorous activity (such as brisk walking or cycling) every day.

Despite the evident benefits of physical activity, the proportion of people and patients adhering to lifestyle recommendation is low. All smoking cessation interventions have proven to be cost-effective.

## 5. Potential Preventive Role of Pharmacological Correction of CV Risk Factors in Neoplastic Diseases

It is well known that oncologic therapies may be dangerous for the heart [5]. In contrast, in CV prevention, an array of pharmacological interventions is employed, each potentially exhibiting pleiotropic effects that may either impede or foster tumor degeneration and progression [99,100,101,102,103]. Insights from cellular and animal models inform human studies, comprising meta-analyses of secondary endpoints derived from clinical trials, administrative databases, observational studies, and case–control investigations (Table 1).

**Beta-blockers:** No substantive evidence indicates that their use correlates with a diminished incidence of ovarian, colorectal, pulmonary, prostatic, or mammary neoplasms [117]. However, beta-blockers may confer protective benefits against melanomas and hepatocellular carcinomas in individuals afflicted with cirrhosis and esophageal varices.

**Calcium antagonists:** The risk of neoplasm development appears to remain neutral among patients undergoing treatment with calcium antagonists. Indications suggest a reduction in ovarian neoplasms [118] and an escalation in pulmonary neoplasms, alongside conflicting data concerning mammary neoplasms.

**Renin–angiotensin system inhibitors:** Epidemiological investigations have yet to establish significant correlations between the administration of angiotensin receptor blockers (ARBs) and ACE inhibitors and neoplastic occurrences. Evidence suggests a reduction in esophageal, pulmonary, prostatic, and colorectal tumors and an elevation in melanomas and renal, pulmonary, and mammary tumors [119,120].

**Sacubitril/valsartan:** Sacubitril/valsartan demonstrates a favorable safety profile and significantly enhances both functional and structural echocardiographic markers. It also reduces N-terminal pro-B-type natriuretic peptide levels and alleviates symptoms in patients experiencing cardiac dysfunction secondary to cancer treatment. Emerging evidence suggests that sacubitril/valsartan may exert cardioprotective effects by mitigating myocardial fibrosis, reducing oxidative stress, and improving endothelial function in oncologic patients. Additionally, recent studies indicate its potential role in modulating inflammatory pathways and attenuating chemotherapy-induced cardiotoxicity, highlighting its promise as a therapeutic strategy for preserving cardiac health in cancer survivors [111].

**Alpha-blockers:** In females, both selective and non-selective alpha-blockers demonstrate no association with the risk of mammary tumors, while in males, the relative risk of developing prostatic neoplasms appears attenuated [121].

**Diuretics:** Various reports suggest that the use of diuretics, particularly thiazides, correlates with neoplasm development, especially mammary, ovarian, renal, colonic, and cutaneous neoplasms [122]. However, such associations may be attributable to initial neoplastic symptoms (reverse causality). Conversely, spironolactone seems linked to a diminished risk of prostatic neoplasms in males and bladder neoplasms in females [123].

**Antiplatelet agents:** Since 1971, numerous studies have posited a salutary effect of aspirin therapy on neoplasm incidence, particularly concerning gastrointestinal, cutaneous, mammary (particularly in hormone receptor-positive/HER2-negative subtypes), prostatic, and uterine–ovarian neoplasms, and lymphomas [114,124,125,126,127,128,129]. Patients who take aspirin regularly have been considered less likely to develop colorectal, esophagus, stomach, hepato-biliary, and pancreatic cancer. On the other hand, no influences on head and neck cancer have been reported.

Such effects are linked to treatment duration (minimum of 5 years) and are partly corroborated by other NSAIDs. Cyclooxygenase (COX)-dependent (whose primary effectors are likely to be the suppression of prostaglandins through irreversible COX enzyme inactivation) and COX-independent mechanisms have been proposed to explain the protective effect of aspirin on colorectal cancer [130].

Novel antiplatelet agents, such as clopidogrel, prasugrel, ticagrelor, and vorapaxar, have been implicated in heightened neoplastic risk [122]. This has been attributed to the dissolution of aggregates between neoplastic cells and platelets, facilitating metastatic dissemination [129], although recent reviews refute this notion [131]. The question remains as to whether dual antiplatelet therapy with aspirin and clopidogrel yields a greater neoplastic incidence compared to aspirin monotherapy [132,133].

**Anticoagulants:** Experimental models have suggested a potential antitumor effect of warfarin, yet epidemiological studies have not yielded definitive data. Nevertheless, a recent extensive Norwegian cohort study underscored a notable reduction in the risk of certain solid tumors (lung, prostate, and mammary) [134]. No specific literature data address the effects of novel oral anticoagulants on neoplasm risk.

**Hypoglycemic agents:** An array of epidemiological studies have delineated an association between metformin use and reduced tumor incidence, though this remains a contentious issue [18,135]. Nonetheless, such associations are confirmed for specific neoplasms, like lung, [136] gastroesophageal, and colorectal cancers [137,138]. Evidence concerning insulin, thiazolidinediones, and sulfonylureas is less robust, while dipeptidyl peptidase-4 inhibitors may exacerbate pancreatic neoplasms [139]. Certain antidiabetic agents, including SGLT2 inhibitors, GLP-1 receptor agonists, DPP-4 inhibitors, and metformin, have been implicated in reducing pancreatic cancer risk through diverse biological mechanisms. Metformin, for instance, is thought to exert anticancer effects by activating the AMPK pathway, reducing insulin resistance, and inhibiting mTOR signaling, which may suppress tumor growth. GLP-1 receptor agonists have demonstrated potential in modulating inflammation and apoptosis, whereas SGLT2 inhibitors may influence metabolic reprogramming and oxidative stress in pancreatic cells. Additionally, DPP-4 inhibitors have been explored for their immunomodulatory properties, which could contribute to a protective effect against malignancy [104]. Emerging evidence suggests that SGLT2 inhibitors may exert antineoplastic properties through multiple mechanisms, including metabolic reprogramming, the reduction of insulin and glucose availability in the tumor microenvironment, and attenuation of inflammation. Preclinical studies indicate that these agents can inhibit cancer cell proliferation and induce apoptosis in various malignancies, such as pancreatic, breast, and prostate cancer. Additionally, SGLT2 inhibition has been linked to reduced oxidative stress and modulation of immune responses, potentially limiting tumor progression. However, further clinical investigations are necessary to elucidate the precise mechanisms and therapeutic implications of SGLT2 inhibitors in oncology. Glucagon-like peptide-1 receptor agonists (GLP-1 RAs), primarily used for glycemic control and weight management in diabetes and obesity, have shown potential in cancer prevention through various mechanisms. These agents modulate insulin secretion, reduce systemic inflammation, and promote metabolic homeostasis, which may contribute to a lower cancer risk. Preclinical and epidemiological studies suggest that GLP-1 RAs can inhibit tumor cell proliferation, induce apoptosis, and suppress angiogenesis in several malignancies, including pancreatic, colorectal, and breast cancer. Additionally, GLP-1 signaling has reduced oxidative stress and improved mitochondrial function, potentially limiting DNA damage and tumor initiation. While emerging data are promising, further clinical trials are needed to confirm the long-term protective effects of GLP-1 RAs against cancer.

**Lipid-lowering agents:** With the advent of statins for CV prevention, concerns regarding their potential neoplastic risk arose [140]. Over time and with widespread usage, apprehensions have been dispelled [122,141,142,143], with certain studies indicating that long-term statin therapy may curtail the incidence of certain neoplasms, like gastric, hepatic, hematological, or prostatic cancers, albeit potentially heightening colorectal, pancreatic, bladder, and pulmonary cancers [99,138,144,145,146] (Table 2). Patients adhering more rigorously to statin therapy stand to derive more significant benefits. Indicated statin use for CV purposes is not contraindicated in individuals at neoplastic risk or those suffering from tumor pathologies, bearing in mind pharmacological interactions and favoring hydrophilic molecules.

As inhibitors of HMG-CoA reductase, statins not only contribute to cancer prevention but also enhance the efficacy of anticancer treatments. Their antineoplastic properties extend beyond cholesterol biosynthesis inhibition, encompassing a range of pleiotropic effects, such as the regulation of angiogenesis, the promotion of programmed cell death and autophagy, the suppression of tumor metastasis, and the modulation of the tumor microenvironment. These mechanisms suggest a potential role for statins as adjunctive agents in oncology, though further clinical investigations are required to fully establish their therapeutic impact in cancer management [112].

Subsequent research quelled initial apprehensions about the potential influence of ezetimibe on neoplastic development [122], even in patients with markedly low LDL cholesterol levels in the IMPROVE-IT study [177]. Fibrates exhibited a neutral effect on tumor incidence [178].

Proprotein convertase subtilisin/kexin type 9 (PCSK9) inhibitors, initially developed for lipid-lowering therapy in patients with hypercholesterolemia, were recently investigated for their potential role in cancer prevention [179]. PCSK9 plays a crucial role in cholesterol metabolism by regulating low-density lipoprotein receptor (LDLR) degradation; however, emerging evidence suggests that it may also influence tumor progression through its effects on lipid homeostasis, inflammation, and immune modulation. Preclinical studies have indicated that PCSK9 inhibition could enhance antitumor immunity by increasing LDLR expression on immune cells, thereby improving lipid metabolism within the tumor microenvironment [179]. Additionally, some research suggests that PCSK9 inhibitors may reduce cancer cell proliferation by altering cholesterol availability, which is essential for membrane synthesis and cellular signaling in rapidly dividing tumor cells [105]. Despite these promising findings, further clinical trials are needed to determine whether PCSK9 inhibitors confer a direct protective effect against cancer and better understand the underlying mechanisms involved in their potential anticancer properties [179]. Inclisiran, a small interfering RNA (siRNA) therapeutic agent that targets proprotein convertase subtilisin/kexin type 9 (PCSK9) synthesis, has revolutionized lipid-lowering therapy by providing sustained reductions in low-density lipoprotein cholesterol (LDL-C). Beyond its CV benefits, emerging research suggests that inclisiran may have potential implications in cancer prevention [180]. PCSK9 has been implicated in tumor progression through its role in lipid metabolism, immune evasion, and inflammatory signaling [105,181]. By inhibiting PCSK9 production at the hepatic level, inclisiran may enhance immune surveillance by increasing low-density lipoprotein receptor (LDLR) expression on immune cells, thereby promoting lipid homeostasis in the tumor microenvironment [180]. Additionally, the long-term reduction of systemic LDL-C levels may influence cancer cell proliferation, given the critical role of cholesterol in membrane synthesis and intracellular signaling [179,182]. While preclinical studies suggest a link between PCSK9 inhibition and reduced tumor growth, robust clinical trials are needed to clarify inclisiran’s potential role in oncological prevention and its broader impact on cancer-related metabolic pathways [183,184,185].

The coexistence of shared risk factors and potential drug effects for CV prevention presents a unique avenue for enhancing population health. The use of generally inexpensive and extensively studied drugs for neoplasm prevention (pharmacological repositioning) constitutes a viable alternative to the pursuit of costly antitumor medications. Inconsistencies across study findings likely stem from the biological diversity of various neoplasms, population characteristics, unidentified mechanisms, confounding factors, publication biases, and temporal dynamics [186]. For instance, individuals undergoing treatment with CV prevention drugs may demonstrate heightened lifestyle awareness and a greater propensity for oncological screenings. Consequently, observational studies may not elucidate whether an association is causal, and definitive conclusions may prove elusive, potentially engendering unwarranted optimism among physicians and patients. The extant gap in medical research underscores the imperative for intervention studies. Regrettably, such drugs are often generic, and the industry evinces scant motivation to conduct trials. However, such endeavors should be regarded as high-priority research initiatives given their potential to save lives on a monumental scale.

## 6. Lifestyle, Risk Factor Correction, and Prevention of CV Complications and Tumor Recidivism in Neoplastic Patients

Upon receiving a cancer diagnosis, however, many patients tend to adjust their lifestyle, particularly if undergoing antineoplastic treatments causing asthenia (due to marked anemia) or neurological/psychological disturbances [187]. Therefore, a cancer diagnosis may present an opportune moment to encourage patients to modify their lifestyles, encompassing smoking habits, dietary choices, and physical activity. Many smokers quit spontaneously. The fear of treatment side effects and, for cured patients, disease recurrence render patients more receptive to advice regarding nutrition and lifestyle; patients themselves frequently seek such guidance [188,189,190]. Physical activity proves beneficial in counteracting the adverse effects on CV function associated with the tumor and/or antineoplastic therapies, such as tachycardia tendency, reduced heart rate variability, and alterations in blood pressure. It enhances quality of life by alleviating anxiety, sleep disturbances, pain, and fatigue [191,192]. While the role of physical activity in primary neoplasm prevention is increasingly recognized, adopting a healthy lifestyle in recent years has emerged as a means of preventing recurrences, complementing adjuvant therapies. In certain tumor types, maintaining an active lifestyle, mainly engaging in scheduled and consistent physical activity, has been demonstrated to reduce recurrence risk and improve disease prognosis [193,194,195]. Multiple studies on women engaging in physical activity before and after breast cancer diagnosis have shown a 34% reduction in tumor-related mortality, a 41% reduction in all-cause mortality, and a 24% reduction in neoplasm recurrences [196]. Similarly, a reduction of 40–60% in colon cancer-related mortality and >20% in all-cause mortality has been observed due to physical activity post-diagnosis [195]. Naturally, the benefit obtained correlates with the frequency and intensity of physical exercise [197]. There is unanimous consensus among oncologists that physical activity should be encouraged in patients with current or past tumors; strategies to promote adherence to this advice vary and involve personalized programs, reinforcement techniques, and transitioning from supervised programs to patient self-management [198].

Although robust experimental evidence in humans is lacking (though promising animal studies exist), a diet rich in flavonoids—which possess iron-chelating and antioxidant properties—is recommended for patients undergoing anthracycline therapy to mitigate its toxicity. A highly varied diet rich in fruits, vegetables, and whole grains is certainly advisable for oncology patients; however, a vegan diet should be discouraged, as it may cause vitamin B12 deficiency and secondary hyperhomocysteinemia. Self-prescription of “natural” treatments or supplements often advertised on websites, books, or magazines dedicated to “alternative therapies”, not validated by any scientific study, should also be discouraged.

Various antineoplastic treatments, such as androgen blockade used in prostate cancer therapy (for testosterone deficiency), head radiation therapy (the hypothalamus and pituitary are sensitive to radiation, which can cause growth hormone deficiency), neck radiation therapy (for thyroid hormone deficiency), whole-body radiation therapy for stem cell transplantation in hematologic neoplasms, pelvic radiation therapy, and hormonal therapy for breast cancer (with antiestrogenic action), are iatrogenic factors associated with metabolic syndrome [199]. To counterbalance this, various supplements have been tested (flavonoids, selenium, vitamin E, omega-3 and omega-6 fatty acids, herbal nutraceuticals), but have not shown efficacy. Foods rich in soy, selenium, and vitamin E do not seem effective in reducing LDL cholesterol levels and may interfere with some antitumor treatments [200,201]. There are no data suggesting the use of high-dose selenium, zinc, or vitamin supplements.

Finally, there is no definitive certainty that the use of aspirin, metformin, and statins is associated with better prognosis in patients with confirmed neoplasms [99,101,103,133,135,139,202], although the results of some randomized studies are awaited [203].

Recent advancements in cardiac imaging have significantly enhanced the capability for early detection and monitoring of cardiovascular complications in cancer patients. Among these new tools, speckle tracking echocardiography (STE) has emerged as a promising technique. This angle-independent imaging modality enables the precise quantification of myocardial deformation properties, offering a more detailed assessment of myocardial function than traditional echocardiographic methods. Unlike conventional echocardiographic techniques, STE can detect subtle changes in myocardial strain, allowing for early recognition of myocardial dysfunction [5,204].

In the context of oncological care, STE has proven to be particularly valuable in detecting subclinical myocardial dysfunction in patients undergoing antineoplastic treatments. These therapies, including chemotherapy and radiation, are known to be associated with cardiotoxicity, which can lead to heart failure if not appropriately managed. Multiple studies have demonstrated that STE can identify early myocardial dysfunction in cancer patients before clinical symptoms or conventional imaging methods, such as echocardiography or magnetic resonance imaging (MRI). This early detection allows clinicians to implement cardioprotective interventions, including pharmacological therapies, before the onset of overt HF, thereby potentially mitigating the long-term CV consequences of cancer treatment [5,204].

Moreover, STE has been shown to be a useful tool in providing prognostic insights, as longitudinal monitoring of myocardial strain changes may aid in predicting the risk of developing significant cardiac failure. Given the growing use of antineoplastic therapies and the recognized risk of cardiotoxicity, the integration of STE into routine oncological care is warranted. Due to its non-invasiveness, combined with its ability to detect early and subtle myocardial abnormalities, STE should be considered an essential tool for timely management in cancer patients, ultimately improving both short- and long-term patient outcomes [5,204].

## 7. Future Perspectives

Before starting chemotherapy, risk factors must be carefully assessed, and risk stratification should be performed to identify patients at higher risk of cardiovascular complications [205]. These risk factors must also be monitored throughout and after cancer therapy, with the results being systematically shared within the multidisciplinary team responsible for patient surveillance [206]. Effective communication of these findings to both patients and their families is crucial to ensure adherence to preventive strategies. This comprehensive approach is essential to mitigate cancer therapy’s cardiovascular effects and optimize long-term outcomes. Further studies are needed to accurately weigh the risk associated with each factor and identify new cardiotoxicity predictors, ultimately aiming for safer cancer treatments. Integrating artificial intelligence (AI) into predictive analytics represents a significant advancement in this field [207]. AI-driven algorithms can analyze large-scale patient data, detecting subtle patterns indicative of increased cardiovascular risk and enabling early interventions [208]. Machine learning models are being developed to personalize treatment strategies, optimizing therapeutic efficacy while minimizing adverse effects [207]. Additionally, AI-assisted imaging techniques and real-time monitoring via wearable devices may facilitate the early detection of cardiotoxicity, allowing for timely modifications in treatment regimens [207,209]. Despite these advancements, many developing countries continue to face obstacles in implementing effective preventive and management strategies due to economic imbalances, inadequate healthcare infrastructure, and low levels of patient education. These barriers contribute to persistent risk factors, such as smoking, unhealthy dietary habits, and lack of adherence to medical recommendations. A more individualized approach is needed to address these challenges, incorporating frequent medical check-ups, patient education on lifestyle modifications, and enhanced accessibility to specialized care [210]. Ultimately, a multidisciplinary effort is essential to integrate innovative technologies and preventive measures, ensuring that cancer therapies can be administered with improved safety and reduced cardiovascular risks.

## 8. Conclusions

The role of primary healthcare providers and cardiologists in cancer prevention should be expanded, emphasizing the importance of integrating oncological risk reduction strategies into routine CV care. A particular focus should be placed on individuals with a family history of cancer, as they may benefit most from targeted prevention efforts. Education and awareness programs should highlight the significance of modifiable risk factors, such as smoking cessation, maintaining a healthy diet, and engaging in regular physical activity, all of which contribute to both primary and secondary prevention of CVDs and malignancies.

While lifestyle modifications play a crucial role, pharmacological interventions should not be overlooked in patients at high CV risk, including those predisposed to or already diagnosed with cancer. Implementing evidence-based medical therapies in CV prevention should be carefully considered to balance benefits and potential risks, ensuring optimal patient outcomes. A multidisciplinary approach involving oncologists, cardiologists, and primary care physicians is essential to developing comprehensive prevention strategies addressing CV and cancer-related health concerns. Future research should continue exploring the intersection between CV and oncological care, fostering a more integrated disease prevention and management model.

## Figures and Tables

**Table 1:** The protective role of CV drugs in cancer prevention.

Drug Class	Potential Role in Cancer Prevention	Mechanism of Action	Evidence/Findings
**SGLT2 Inhibitors (e.g., empagliflozin, dapagliflozin) (Various dosages)** **(2018–2023)**	Antineoplastic properties, including reduced pancreatic, breast, and prostate cancer risks.	Modulates metabolic reprogramming, reduces glucose and insulin availability in tumor microenvironment, reduces oxidative stress.	Preclinical studies suggest inhibition of cancer cell proliferation, induction of apoptosis, and reduced oxidative stress [104]. Clinical trials are needed for validation.
**PCSK9 Inhibitors** (Various dosages) (2015–2023)	Potential reduction in cancer risk, especially related to tumor growth and immune response.	Modulates lipid metabolism, enhances immune cell function by increasing LDL receptor expression, may reduce cancer cell proliferation.	Early preclinical studies suggest potential anticancer effects through immune modulation and altered cholesterol availability in tumors [105]. Clinical trials are needed for further investigation.
**GLP-1 Receptor Agonists (e.g., liraglutide, semaglutide)** (Various dosages) (2016–2024)	Potential reduction in colorectal, pancreatic, and breast cancer risks.	Modulates insulin secretion, reduces systemic inflammation, promotes apoptosis, inhibits tumor cell proliferation, and improves mitochondrial function.	Epidemiological and preclinical data suggest these agents could inhibit tumor cell growth and reduce oxidative stress, potentially lowering cancer incidence [106]. Further clinical studies are needed to confirm these findings.
**Beta-Blockers** **(Various dosages)** **(1990–2015)**	Protective effects against melanomas and hepatocellular carcinoma, particularly in patients with cirrhosis and esophageal varices.	Reduces sympathetic nervous system activity, may inhibit cancer cell proliferation.	No strong evidence for a reduction in incidence of common cancers (e.g., ovarian, colorectal) [107]. Potential protective effect seen in specific cancers (e.g., melanoma) [108].
**Calcium Antagonists** **(Various dosages)** (1985–1998)	Neutral or slightly protective role in ovarian neoplasms, but possible risk increase in pulmonary cancers.	Block calcium channels, may affect cellular growth and survival.	Evidence indicates no significant effect on overall cancer incidence, though some studies suggest reduced ovarian and increased pulmonary cancer risks [109].
**Renin–Angiotensin System Inhibitors (e.g., ACE inhibitors, ARBs)** **(Various dosages)** **(1991–2012)**	Reduction in esophageal, pulmonary, prostatic, and colorectal cancers, with potential increase in melanomas and kidney cancers.	Modulates blood pressure, impacts cell growth, and inhibits tumor progression via antiangiogenic effects.	Mixed results, with some studies showing a protective effect, while others suggest potential harm in certain cancers (e.g., kidney, melanoma) [110].
**Sacubitril/Valsartan** **(Various dosages)** (2016–2020)	Potential cardioprotective effects in cancer survivors, reducing chemotherapy-induced cardiotoxicity.	Enhances endothelial function, reduces myocardial fibrosis, modulates inflammation.	Shows promise in reducing heart damage caused by cancer treatment, with some evidence suggesting protective effects against tumor progression [111].
**Statins** **(Various molecules and dosages)** **(2001–2023)**	Possible reduction in gastric, hepatic, hematological, and prostate cancers; no significant effect on colorectal, pancreatic, bladder, or lung cancers.	Inhibits HMG-CoA reductase, which is involved in cholesterol biosynthesis, modulates angiogenesis, and promotes apoptosis.	Long-term use linked to reduced incidence in some cancers [112]. Their pleiotropic effects may help suppress tumor metastasis and improve cancer treatment efficacy [113].
**Aspirin** **(100–325 mg)** (2007–2016)	Potential reduction in gastrointestinal, breast (hormone receptor-positive), and prostatic cancers.	COX inhibition, suppresses prostaglandins, potentially reducing cancer cell growth.	Long-term use (5+ years) shown to reduce risks in certain cancers, especially colorectal and gastric cancers [114].
**Diuretics** **(Various dosages)** **(1990–2013)**	Possible correlation with increased cancer risk, particularly in mammary, ovarian, and renal cancers.	Alters fluid balance, impacts renal function.	Potential link to cancer risk, but likely due to reverse causality or confounding factors [115]. Spironolactone may reduce prostate cancer risk in men and bladder cancer in women [116].

**Table 2:** Influence of statin therapy on the incidence of various types of cancer.

Cancer Type	Effect	Study Design	Sample Size	Author
**Colorectal**				
	≈	Case–control	4606	Pottegard A [147]
	↓	Prospective cohort	783	Liu JC [148]
	↓	Case–control	22,163	Mamtani R [149]
	↓	Meta-analysis	>100,000	Lytras T [150]
**Skin**				
	≈	Meta-analysis	>11,000	Li X [151]
	≈	Retrospective cohort	1099	Jagtap D [151]
	↑	Case–control	> 40,000	Arnspang S [152]
	↑	Meta-analysis	57,004	Yang K [153]
**Brain**				
	↓	Case–control	517	Ferris J [154]
**Endometrial**				
	≈	Case–control	5382	Sperling CD [155]
	≈	Meta-analysis	9517	Yang J [156]
**Liver**				
	≈	Case–control	2877	Friedman GD [157]
	↓	Meta-analysis	35,756	Shi M [158]
	↓	Meta-analysis	10,993	Zhou YY [159]
**Esophageal**				
	↓	Meta-analysis	1057	Thomas T [160]
	↓	Systematic review	35,214	Alexandre L [161]
**Non-Hodgkin Lymphoma**				
	↓	Case–control	1715	Cho SF [162]
**Breast**				
	≈	Meta-analysis	121,399	Islam MM [163]
	↓	Prospective cohort	18,769	Ahern TP [164]
	↓	Retrospective cohort	15,718	Anothaisintawee T [165]
	↑	Case–control	1582	McDougall JA [166]
	≈	Prospective cohort	7430	Desai D [167]
	↓	Prospective cohort	4216	Boudreau DM [168]
**Multiple Myeloma**				
	↓	Case–control	2532	Epstein MM [169]
**Ovarian**				
	≈	Case–control	4103	Baandrup L [170]
**Pancreatic**				
	↓	Case–control	704	Kho P [171]
	↓	Retrospective cohort	2341	Chen MJ [148]
**Lung**				
	↓	Prospective cohort	1225	Liu JC [148]
	≈	Meta-analysis	38,013	Tan M [172]
**Prostate**				
	≈	Prospective cohort	9457	Platz E [173]
	↓	Meta-analysis	61,958	Bansal D [174]
**Kidney**				
	≈	Meta-analysis	870	Zhang XJ [175]
**Biliary**				
	↓	Case–control	3174	Peng YC [176]

Symbols: ≈: neutral effect; ↑: increase; ↓: reduction.
